# A single, shared origin for all three coronary arteries from the right coronary cusp: a case report

**DOI:** 10.1186/s13256-020-02422-9

**Published:** 2020-07-10

**Authors:** Zeid Nesheiwat, Joseph Eid, Ronak Soni, Paul Harnish, Ebrahim Sabbagh, Ehab Eltahawy

**Affiliations:** 1 Department of Internal Medicine, The University of Toledo Medical Center, Toledo, OH USA; 2 Department of Cardiovascular Medicine, The University of Toledo Medical Center, Toledo, OH USA

**Keywords:** congenital, anomaly, coronary computed tomographic angiogram, case report, sudden death, unroofing, case report

## Abstract

**Background:**

Anomalous coronary arteries occur in less than 1% of the population and have been implicated in sudden cardiac and exercise-related death. The most common variant involves the left circumflex artery arising from a separate ostium than the left coronary artery. This case demonstrates a rare variation in which all three coronary arteries arise from a shared, single, ostium originating from the right coronary cusp.

**Case presentation:**

We report the case of a 63-year-old Caucasian man with a history of myocardial infarction, congestive heart failure, and atrial fibrillation who presented for syncope. Inpatient ischemic workup, including coronary angiography, demonstrated a rare coronary anomaly which included all three coronary arteries arising from a shared, single, ostium originating from the right coronary cusp. Our patient was treated conservatively with an option for coronary bypass if symptomatic.

**Conclusion:**

Surgical management is indicated in high-risk patients, but the optimal management for a nonmalignant, shared origin for all three coronary arteries has not been explored in detail.

## Introduction

Coronary artery anomalies (CAA) are caused by developmental malformations, typically congenital, within the coronary arteries. Most often, these abnormalities are benign and related to origin and/or location of these arteries and it is estimated that less than 1% of the population possess CAA, the most common variant being a separate origin of the left anterior descending (LAD) and left circumflex artery (LCX) occurring in approximately 0.4% of cases, followed by the LCX arising from the right coronary artery (RCA) in approximately 0.37% of cases [1].

Most CAA are asymptomatic and found incidentally later in life through cardiac imaging. However, high-risk CAA do exist that can lead to severe debility and sudden cardiac death [2]. It is important to evaluate all CAA for “malignant” courses that may be high risk. Coronary computed tomographic angiography (CCTA) is considered the ideal imagining modality.

## Case presentation

**Fig. 1:**
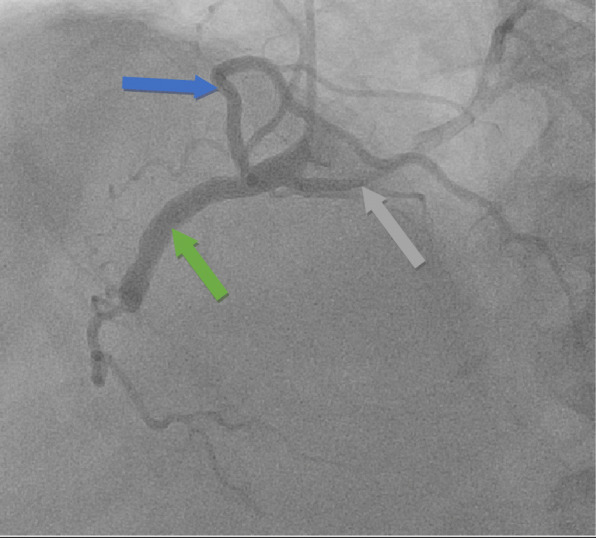
Left heart catheterization revealing anomalous coronary arteries all arising from the right coronary cusp. *Green arrow*: right coronary artery. *Blue arrow*: left anterior descending artery. *Gray arrow*: circumflex artery

**Fig. 2:**
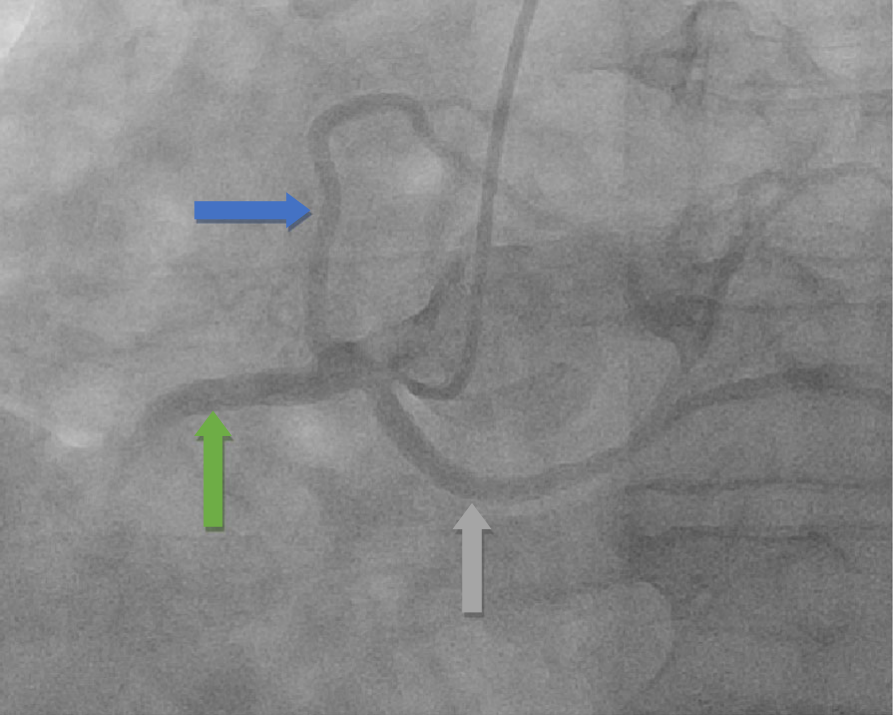
Left heart catheterization revealing anomalous coronary arteries all arising from the right coronary cusp. *Green arrow*: right coronary artery. *Blue arrow*: left anterior descending artery. *Gray arrow*: circumflex artery

**Fig. 3:**
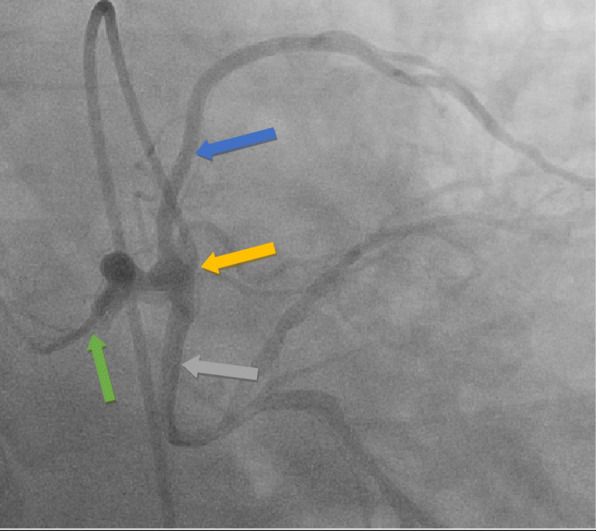
Left heart catheterization revealing anomalous coronary arteries all arising from the right coronary cusp. *Green arrow*: right coronary artery. *Blue arrow*: left anterior descending artery. *Gray arrow*: circumflex artery. *Yellow arrow*: origin of all three coronary arteries

**Fig. 4:**
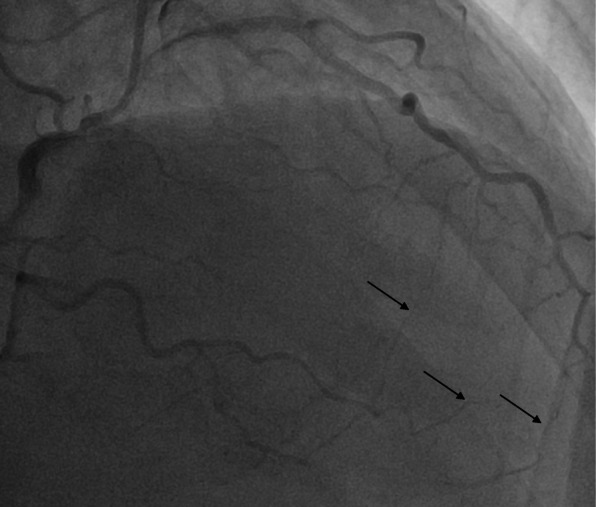
Left heart catheterization revealing right coronary artery collaterals from the septal branches of the left anterior descending to the posterior descending artery (*black arrows*)

**Fig. 5:**
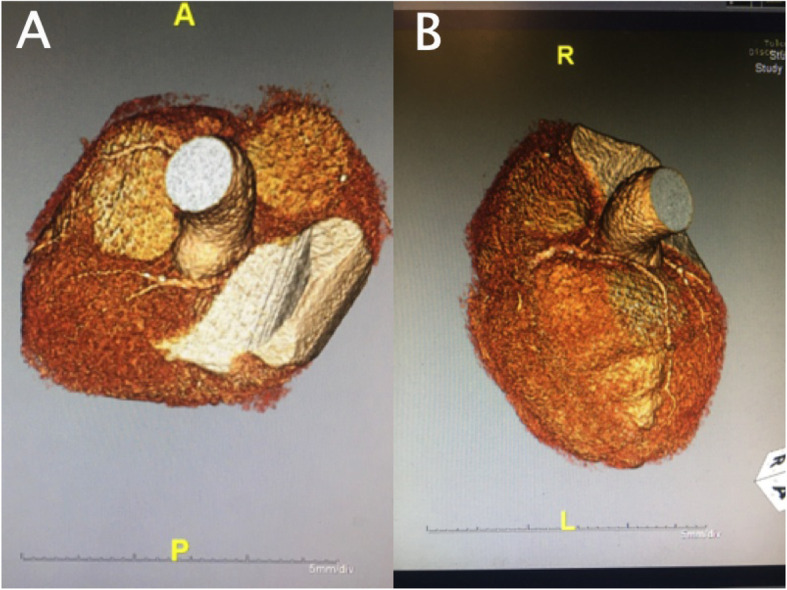
Two views (**a** and **b**) from CCTA imaging revealing the left anterior descending artery originating posteriorly and coursing between the aorta and left atrium. *CCTA* coronary computed tomographic angiography

Our patient is a 63-year-old Caucasian man with a past medical history of recent myocardial infarction (MI) status post unsuccessful percutaneous coronary intervention, heart failure with reduced ejection fraction (25–30%), coronary artery disease, and atrial fibrillation who presented to our institution with a 3-day history of syncope without chest pain or dyspnea. Initial vital signs reveal tachycardia and hypotension (lowest recorded 85/64). Initial testing revealed new-onset atrial fibrillation with rapid ventricular response, negative troponin, chest X-ray revealing cardiomegaly, and negative computer tomography of the brain. Subsequent cardiac catheterization revealed a single ostium shared by all three coronary arteries (right coronary artery, LCX, and LAD) all originating from the right coronary cusp with the left anterior descending artery originating posteriorly and coursing between the aorta and left atrium (Figs. 1, 2 and 3). In addition to an occluded mid RCA, distal disease of the LAD and severe disease of the superior branch of the obtuse marginal artery of the LCX were noted. The occluded RCA appeared to have left-to-right collaterals from the septal branches of the LAD to the posterior descending artery (PDA) (Fig. 4). Next, a CCTA was performed to evaluate the artery for a ‘malignant course” defined as an anomalous origin of the coronary artery with subsequent course between the aorta and pulmonary trunk. It is considered malignant as the affected coronary artery can get compressed between the aorta and pulmonary trunk, specifically during exercise, and can produce angina or in worst cases, sudden cardiac death. Our patient was found to have a non-malignant course (Fig. 5). Our patient was evaluated by cardiothoracic surgeons as well as other interventionalists with the decision to treat conservatively, with the option for coronary artery bypass graft (CABG) with or without repositioning of the artery if he remained symptomatic (Table 1). Our patient ultimately underwent successful dual-chamber implantable cardioverter-defibrillator (ICD) placement several months later secondary to ischemic cardiomyopathy with reduced ejection fraction (25–30%) after maximally tolerated medical therapy. Of note, our patient did experience a recent MI 23 days prior to this admission. A cardiac catheterization at that time did show a completely occluded mid RCA with unknown chronicity of the lesion. However, it appeared to be subacute or chronic as the vessel was unable to crossed by the guidewire. Anomalous origin of his coronary arteries was noted at that time. As no percutaneous coronary intervention (PCI) could be performed, our patient was treated medically and later discharged home.

**Table 1:** Timeline of events during hospitalization

Admission day (Day 1)	Patient admitted for syncope. Initial workup was negative.
Day 2 of admission	Cardiac angiography performed showing the coronary anomaly. Same day CCTA showing “non-malignant” LAD course.
Day 3 of admission	Patient was discharged with strict follow-up with his cardiologist with an option for CABG if symptomatic in the future.

*CCTA* Coronary computed tomographic angiography, *LAD* Left Anterior Descending Artery, *CABG* Coronary Artery Bypass Graft

## Discussion

Coronary artery anomalies are extremely rare. Less than 1% of the general population is estimated to have CAA, the majority of which involve different ostia of the LCX and when the LAD and LCX artery arise from the right coronary ostium [1]. This case represents all three major coronary arteries originating from a single ostium, the right coronary cusp with a non-malignant course. There are no guidelines addressing the optimal management approach for this anomaly. Although most CAA are asymptomatic and benign, certain anomalies have been related to sudden cardiac death as well as exercise-related death, typically in the young. These unfortunate circumstances have been more pronounced with high-risk anomalies, most commonly involving the left main coronary artery originating from the right coronary cusp. Other high-risk anomalies include a coronary artery coursing between the aorta and pulmonary artery, acute-angle take off, and ostial abnormalities such as slit-like orifice and beak-shaped orifice [3]. It is critical to identify the course of the arteries as well as evaluate the geometry of the orifice. Diagnosis is commonly established incidentally during routine cardiac testing, but CCTA is considered ideal for geometric evaluation.

Surgical management is the mainstay of treatment for high-risk patients, if indicated. The goal of surgery is to limit the risk of myocardial ischemia. According to the most recent surgical guidelines for treatment of anomalous coronary arteries, unroofing is indicated for vessels with extensive intramural course. Either ostial translocation (reimplantation) or ostioplasty are indicated for vessels with very short intramural course. Bypass grafting is indicated if the anomaly is accompanied by atherosclerotic narrowing or if other reconstructive interventions have failed. With the considerable advancement of percutaneous coronary intervention, treatment has now been extended to include stenting of the anomalous vessels [4]. There is limited investigation into this particular anomaly arising from the same ostium at the right coronary cusp. Despite all the options, there has been considerable variation in the management of anomalous coronary arteries as a whole, thus establishing the need for further investigation into a more standardized approach.

## Conclusion

Patients with CAA should undergo further investigations to delineate the origin and course of the arteries involved; CCTA is usually helpful in such cases. A non-malignant, single, shared origin of all three coronary vessels is a very rare entity. There are no official guidelines addressing the optimal management approach.

### Consent

Written informed consent was obtained from the patient for publication of this case report and any accompanying images. A copy of the written consent is available for review by the Editor-in-Chief of this journal.

## Data Availability

Not applicable.
